# Particle Nanoarchitectonics for Nanomedicine and Nanotherapeutic Drugs with Special Emphasis on Nasal Drugs and Aging

**DOI:** 10.3390/biomedicines11020354

**Published:** 2023-01-26

**Authors:** Tariq Aziz, Abad Ali Nadeem, Abid Sarwar, Ishrat Perveen, Nageen Hussain, Ayaz Ali Khan, Zubaida Daudzai, Haiying Cui, Lin Lin

**Affiliations:** 1 School of Food & Biological Engineering, Jiangsu University, Zhenjiang 212013, China; 2 Food and Biotechnology Research Centre, Pakistan Council of Scientific and Industrial Research Centre, Lahore 54590, Pakistan; 3 Institute of Microbiology and Molecular Genetics, New Campus, University of the Punjab, Lahore 54590, Pakistan; 4 Department of Biotechnology, University of Malakand, Chakdara 18800, Pakistan; 5 Department of Bioresources and Biotechnology, King Mongkut University of Technology, Bangkok 10140, Thailand

**Keywords:** angiogenesis, FOLR1, GATA6, nanotherapeutics, nasal drugs, NALT, vascular endothelial growth factor (VEGF)

## Abstract

Aging is a multifunctional physiological manifestation. The nasal cavity is considered a major site for easy and cost-effective drug and vaccine administration, due to high permeability, low enzymatic activity, and the presence of a high number of immunocompetent cells. This review article primarily focuses on aging genetics, physical parameters, and the use of nanoparticles as delivery systems of drugs and vaccines via the nasal cavity. Studies have identified various genes involved in centenarian and average-aged people. VEGF is a key mediator involved in angiogenesis. Different therapeutic approaches induce vascular function and angiogenesis. FOLR1 gene codes for folate receptor alpha protein that helps in regulating the transport of vitamin B folate, 5-methyltetrahydrofolate and folate analogs inside the cell. This gene also aids in slowing the aging process down by cellular regeneration and promotes healthy aging by reducing aging symptoms. It has been found through the literature that GATA 6, Yamanaka factors, and FOLR1 work in synchronization to induce healthy and delayed aging. The role and applications of genes including CBS, CISD, SIRT 1, and SIRT 6 play a significant role in aging.

## 1. Introduction

A decrease in different functions of body organs refers to aging that increases the death risk. Due to the progressive decrease in the reparative and regenerative capacity of tissues and organs with a reduced physiological reserve in response to stress, the process of aging is, thus, linked to the decay of homeostasis [1]. Aging is also related to oxidative damage which involves tissue impairment and immunosenescence or metabolic imbalances which trigger inflammation and inflammasome formation. Changes that are linked with aging can affect the overall appearance of the body including wrinkled skin, grey hair, dryness of the skin, and thin skin whereas some changes affect organs’ efficiency to perform their functions which may result in the development of various diseases. Among all these factors one of the chief aging-related alterations is the dysregulation of the immune response which results in chronic low-level, systemic inflammation known as “inflammaging” [2,3]. Genetic and epigenetic changes, as well as environmental factors, promote and modulate the mechanisms of aging at the molecular, cellular, organ, and system levels which are further characterized by time-dependent patterns of variation driven by the biological clock. According to various research, aging increases the risks of heart disease, hypertension, etc., in humans [4,5,6]. However, since the mid-nineteenth century, the average life expectancy of humans has increased by three months per annum [7]. In the start, it was increased by controlling infectious diseases but later it was improved by treating the diseases that were linked to aging. Nanoparticles exhibit anti-aging properties. They have been extensively reported as a skin-protectant against extrinsic factors. By analyzing genes between different age groups people may find the molecular pathways that can be important in aging. The aging track varies from individual to individual. Some studies have indicated that a correlation in lifespan exists between monozygotic as compared to dizygotic twins [8,9,10]. 

## 2. Biosynthesis and Bioavailability of Nanoparticles

Microorganisms can produce nanoparticles through both intracellular and intercellular biosynthesis. This process can occur in a variety of different microorganisms, making it a versatile method for nanoparticle production. Different types of microbes can control the synthesis of various metallic nanoparticles. Fungi are especially noteworthy due to their high tolerance and ability to bioaccumulate metals, making them ideal for nanoparticle synthesis [11,12,13,14]. Nanoparticles can also be synthesized using hydrophobic polymers which may include sodium alginate, chitosan, gelatin, etc. This technique produces nanoparticles by coacervation caused by the interaction of positive ions formed by the combination of the two aqueous processes, i.e., polymer chitosan and sodium tripolyphosphate [15]. Nanoparticles aid in the bioavailability and delivery of cancer-specific and tissue-targeted drugs. Modification of nanoparticles ensures drug load and delivery to the specific target site [16,17,18]. An insight into the novel synthetic approach for the mitigation of the detrimental effects of the aging process found that a surface-stabilized mechanism mediated by ZnO nanoparticles fuels the aging process. This mechanism involves gradual but irreversible, active adsorption of oxygen which results in the surface-termination process of HO-ZnO which leads toward the reduction of non-radiative recombination and enhances subsequent built-in potential in the adjoining photoactive layer [17,18,19,20,21]. The extracellular matrix (ECM) is degraded by the enzyme matrix metalloproteinase-1 (MMP-1) in a hyperglycemic environment, which may speed up the aging process of the skin. Gallic acid-coated gold nanoparticles (GA-AuNPs) exhibit a higher potential to diminish high-glucose-mediated MMP-1-induced ECM degradation. This mechanism highlights its anti-aging capability [22,23,24,25,26,27,28,29].

## 3. Nanocarriers and Drug Delivery for CNS

Neuroprotective phytochemicals found in medicinal and nutritional plants can have positive effects on the nervous system. Nanotechnology-based therapeutics show tremendous application for treating central nervous system (CNS) disorders [20,21,22]. The therapeutic benefits of neuroprotective phytochemicals are thought to be due to their antioxidant properties [21]. However, the bioavailability of these natural compounds is often limited by the body’s quick metabolism, lack of stability, and inability to penetrate the blood-brain barrier [30,31,32,33,34,35,36]. Curcumin, a polyphenol derived from *Curcuma longa* L., is one of the most popular and well-studied neuroprotective agents. By modulating key biological and pharmacological targets, nanotechnology can produce significant results in terms of symptoms, relief, and disease management. This approach has the potential to revolutionize the treatment of Alzheimer’s, Parkinson’s, and multiple sclerosis. By targeting the underlying causes of these diseases, we may be able to improve the quality of life for patients and their families [30,37,38,39,40]. Polymers for regulated drug release are in the form of hydrophobic dyes which diffuse at a regular rate through silicone tube walls [41,42]. This discovery paved the way for the possibility of using polymeric materials to administer drugs in a controlled way [43,44]. Tumor therapy presents a unique challenge when it comes to delivering therapeutic chemicals directly to the site of the tumor [45,46,47,48]. The confined diffusion area and the potential for inflammatory responses to implants can make it difficult to deliver these compounds effectively. However, recent advances have made it possible to tailor these delivery techniques to better target the tumor site [49,50,51,52,53].

**Figure 1:**
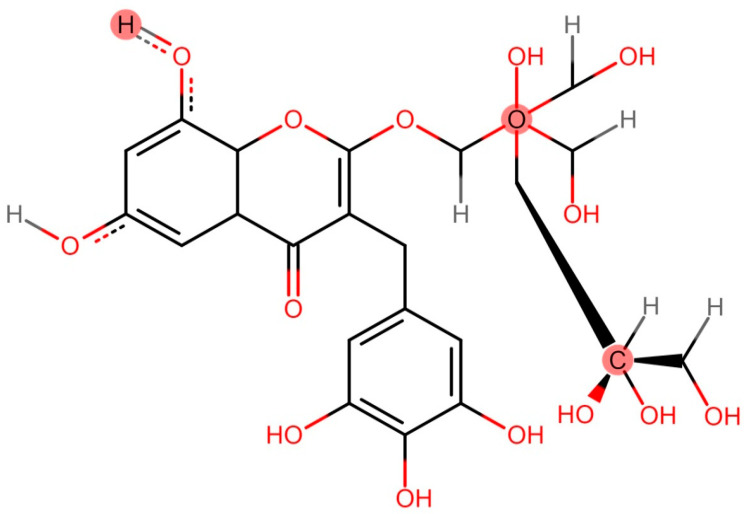
Structural formula of myricetin 3-O-d-galacto-pyranoside (M3G), C_21_H_20_O_13_, which acts as an anti-aging chemical by reducing the activity of aging-related proteins; ERK, JNK, and MAPK [54].

One of the major effective extrinsic components in the skin aging process is UV irradiation or photo-aging. It causes a reduction in the elasticity and strength of skin by damaging the extracellular matrix. The component myricetin 3-O-_-d-galacto-pyranoside (M3G), a flavonol glycoside extracted from *L. tetragonum*, UVA caused changes in (matrix metalloprotenease-1) MMP1. Another factor, activation of (mitogen-activated protein kinase) MAPK, has been observed in pro-inflammatory cytotoxin actions. The application of myricetin 3-O-d-galacto-pyranoside (M3G), had a positive effect on delaying of the aging process. (M3G) blocks the production of chemicals that induce early aging; those are HaCaT keratinocytes and HDFs (Figure 1) [54].

**Figure 2:**
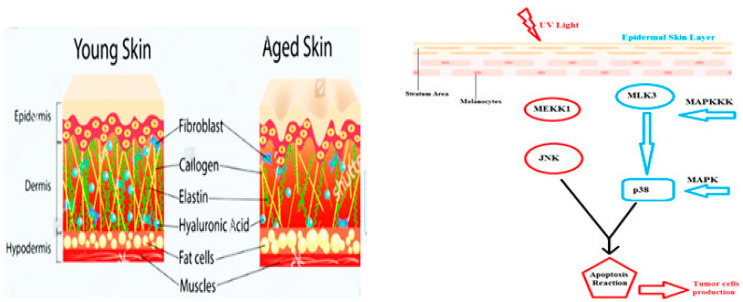
Showing the UV light effect in changing the structure of fibroblast cells which convert normal cell lines into tumor cells via MAPK and JNK pathways [54,55].

Early aging is started by the production of (extracellular signaling kinase) ERK, (c-Jun N-terminal kinase) JNK, and (mitogen-activated protein kinase) MAPK, which can be reduced by the application of (M3G). Three factors of UV have been observed that induced the aging process in the skin, i.e., UVA, UVB, and UVC. The most lateral element of UV radiation is blocked by the ozone layer around the earth’s surface. The other two components, however, may reach the skin, while having properties to penetrate the skin cells, which degrade the upper epidermal layer of the skin by developing wrinkles and activating carcinogenic cells. Damage of fibroblast cells reduces the firmness of skin cells (Figure 2) [54,55].

**Figure 3:**
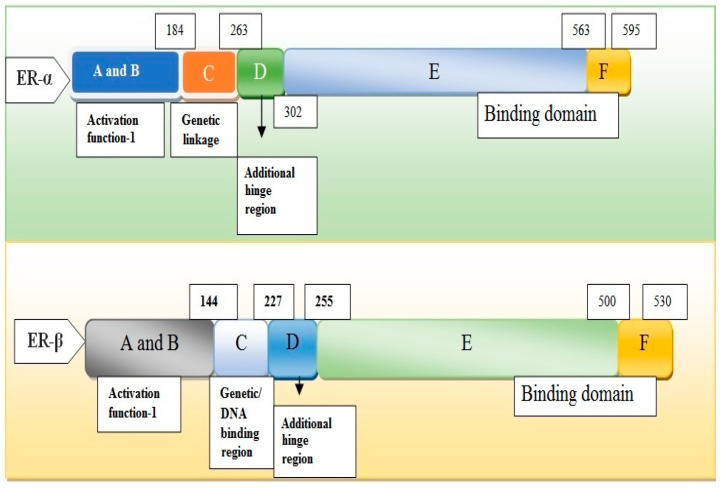
Genetic structure of receptors ER-α and ER-β shows activation regions A and B, genetic linkage region C, hinge region D, Ligand-binding domain E, and binding domain F [57].

Aging is enhanced due to atrophy of skin cells especially the dermis, and epidermis, proliferation of dryness, and wrinkling. Aging enforces a negative effect on fibroblast cells of the skin which adds extra pressure on collagen cells. This may also occur during menopause in women. Menopause needs a balanced level of estrogen inside the body to keep function and structure in shape [56,57,58]. Oophorectomy is a process that causes aging in the body. It destroys the collagen level and fibroblast function in the body. Dermal cells, when degraded, may cause pre-menopause situations. Women from the Asian region of the world are used to having a high level of isoflavones in their routine food intake. This chemical is a major source of skin-degradation phenomena. Genistein aglycone is a type of isoflavone found in our daily food materials and plays a significant role in enhancing the aging reaction in the body (Figure 3). Two receptors ER-α and ER-β have been found to play role in the expressivity of healthy cells in the skin. They restore the damaging cells after oophorectomy [57].

Both have two different regions for their reaction (structural and functional). The transactivation domain of ER-α contains coactivation and co-repression. The hinge region in both receptors determines the conformational changes in the receptors. A modification in the transcriptional role can be achieved by the process of acetylation and methylation. ER-β is found to be weaker towards the corresponding function.

During the aging process, the extracellular matrix starts to degrade while altering the function of fibroblast cells or making them non-functional. The senescence cells cause an irreversible reaction that blocks the proliferation and growth of normal cells and promotes the development of carcinogenic cells with the activation of oncogenes. Still, there is a lack of research that enlightens us about specific genes linked to aging. However, in some studies, it is said that aging affects angiogenesis and impaired angiogenesis is related to heart diseases and cancer. Also, some studies tell us about the role of the FOLR1 (folate receptor 1/folate receptor alpha) gene in the aging process [56,57].

Studies on centenarians have been conducted to determine the specific genes that are in higher proportion in centenarians as compared to the average-aged person. Transcriptional approaches and longitudinal approaches are used in many studies for the understanding of the molecular pathways of aging. A longitudinal study is easy, but the transcriptional approach is more powerful for better understanding. Aging impairs angiogenesis, which results in diseases such as cardiovascular diseases and cancer [58]. The main factor of the angiogenesis process is the VEGF (vascular endothelial growth factor) gene. However, factors and stimuli are also present that affect VEGF and angiogenesis. Some diets such as methylation-restricted diets can increase vascular function, hence delaying aging by enhancing the angiogenesis process. Folate plays an important role in either delayed or early aging depending upon its consumption. Intake of folate increases life span and deficiency of folate results in depression, anxiety, and weakness in elderly people. The FDA (Food and Drug Authority) has recommended a specific dosage of folate. A folate-containing diet includes green vegetables and others. For this purpose, supplements can also be used [59]. Genetics of aging regarding the genes FOLR1, GATA6 (GATA-binding protein 6), CBS (Cystathionine beta synthase), CISD2 (CDGSH iron-sulfur domain-containing protein 2), SIRT1(silencing information 1), and SIRT6 (silencing-information 6) was studied in the current article. This review article aimed to understand aging genetics, physical parameters, and nanotherapeutic applications.

## 4. Studies in Octogenarians for Genetic Analysis of Aging

To determine the genes that are linked to aging, one method is to compare the octogenarians’ genome and the average-aged person’s genome. The ratio of survival to centenarian age in the USA is 1/10,000, while the average life expectancy is seventy-seven years [56]. The probability of surviving to age 100 for centenarian siblings increases 8 to 17-fold as compared to those without centenarian siblings [60]. Centenarians probably have enrichment of the alleles that can affect genes that play an important role in increased life span. In one study on 137 centenarians in the USA, it was shown that there was a link between microsomal transfer protein and the life expectancy of humans, but these data may be different in populations of other countries [61].

In some studies that were conducted on elderly people, an allele that increases the probability of diseases including Alzheimer’s disease and cardiovascular diseases is E4 of apolipoprotein-E. This allele has a lower proportion in centenarians which suggests that a person with a higher proportion of this allele has a lower life expectancy as compared to those who have a lower value [62]. On the other hand, there is a higher proportion of E2 alleles in centenarians which suggests that it may have a protective role against age-related diseases [62]. In some studies, it has been found that long telomere lengths are linked with longer life span, and protection from age-related diseases, while in others it has been found that change in insulin/insulin-like signaling pathway genes also increases life expectancy in Caenorhabditis elegans, drosophila, and mice. FoxO (Forkhead box O) is activated by decreasing insulin-growth -actor signaling, which increases the life expectancy of these organisms [63].

### 4.1. Physiological Aging

Other than centenarian studies, one method is to analyze physiological aging, which can be measured easily with the help of indexes of biomarkers found by measuring the process of changes in tissue with chronological age. These biomarkers can also be used to determine if a person’s physiological age is more or less than his chronological age. Global research on ageing has discovered various alleles of genes linked to the ageing process, i.e., sardiNIA, BLSA, and InCHIANT, in populations from around the world [64].

### 4.2. Transcriptional Approach for Studies of Aging

One method to determine the molecular basis of aging is scanning the genome using DNA microarrays to find the genes whose expression varies with age. These variations in the level of gene expression cause change in vital functions of organs in aging. As aging has a polygenic nature, the transcriptional approach is powerful in studies of aging [65]. 

### 4.3. Aging Affects Angiogenesis

**Figure 4:**
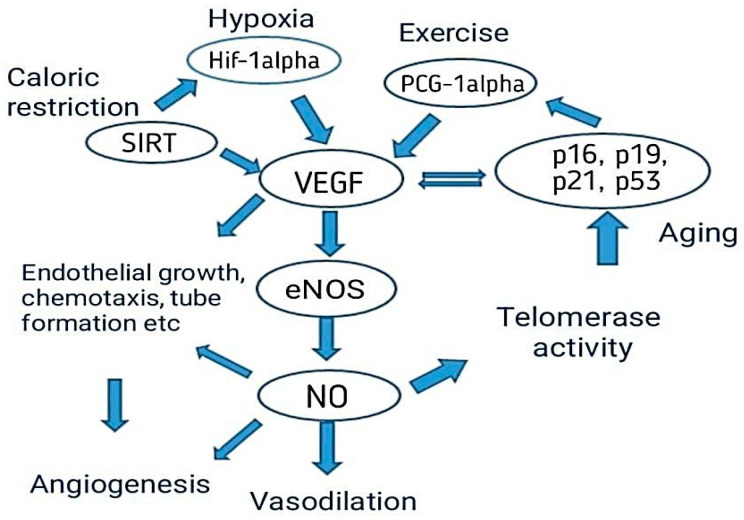
Factors involved in angiogenesis: exercise; hypoxia; caloric restriction; exogenous delivery of VEGF, SIRT, and Hif-alpha, which help in aging reduction [65,66].

Aging increases the risk of cardiovascular diseases and other neurogenic disorders. The main cause of cardiovascular diseases is impaired angiogenesis, and the other cause is endothelial dysfunction. Both increase the prevalence of cardiovascular diseases. Angiogenesis slows the vital adaptive response against physiological change. Angiogenesis is also a repair process after an injury [66]. By understanding the foundation of impairment of angiogenesis by aging, we can manage cardiovascular diseases. Therapeutic studies for heart diseases include the regulation of angiogenesis and recovery from any injury in an organ that needs blood-vessel growth. In elderly people there is a decrease in angiogenesis reacting to ischemia. This is mainly caused by a decrease in Hif-1a activation and expression of VEGF, which decreases the endothelial migration, proliferation, and sprouting and results in reduced EOC number and function [65,66] as shown in Figure 4.

Delivering the angiogenic agents (VEGF, SIRT, Hif-alpha, etc.) exogenously could overcome the decreased molecular response against ischemia in elderly people. Angiogenesis is restored in old animals by exogenous delivery of VEGF-A protein or activating the VEGF-A transcriptionally. This treatment also has other benefits for elderly people. However, clinical tests on the exogenous administration of angiogenic agents have failed. These results highlight the challenges of other risk factors that affect angiogenesis. Other risk factors that affect angiogenesis are obesity and smoking, which are linked to shorter telomeres; on the other hand, exercise protects a person from the risk of cellular aging that is telomere-length dependent [67].

### 4.4. Role of VEGF to Delay Aging

A molecule called vascular endothelial growth factor (VEGF) promotes blood vessel expansion. According to a recent study published in *Science*, physiological aging in numerous organs may be triggered by insufficient VEGF signaling in old mice. The researchers showed that “old mice” with a slight rise in circulatory VEGF could reduce age-related symptoms and increase life expectancy. Every bodily cell depends on blood vessels (BVs) to provide oxygen, other blood-borne nutrients, and, in some conditions, endothelial-derived paracrine factors. The vascular system ages just like other organ systems, which decreases the efficiency of organ functions. It has been postulated that vascular aging is an upstream, founding element in organismal aging given the importance of BVs to organ homeostasis; however, there is little experimental evidence to support this claim. Both small and big vessels are affected by vascular aging, with the latter being characterized by capillary rarefaction, or age-related failure to maintain enough microvascular density (MVD). Vascular endothelial growth factor (VEGF), whose hypoxia inducibility continuously works to replace damaged vessels and match vascular supply to tissue requirements, is a critical homeostatic mechanism that prevents MVD decline. It is still uncertain why VEGF stops functioning properly as a person grows older [68]. It is anticipated that impaired vascular function will disturb organ homeostasis in ways that promote the emergence of age-related illnesses and frailties. Therefore, preventing important aspects of vascular aging may be a good strategy for its relief. We investigated whether maintaining a level of VEGF similar to the level present in young and healthy people could prevent capillary loss and its consequences. VEGF-signaling insufficiency underlies insufficient vascular supply in aging. Based on the hypothesis that impaired vascular function is an upstream cause of multiple organ failure, it is hypothesized that its correction could provide thorough neuroprotection [69]. Longitudinal monitoring showed that VEGF signaling was drastically decreased in numerous important organs, even though VEGF production was not significantly decreased during mouse aging. This was linked to an increase in soluble VEGFR1 (sVEGFR1) production and its ability to act as a VEGF trap due to a shift in alternative splicing of VEGFR1 mRNA linked with aging. Adeno-associated virus (AAV)-assisted VEGF transduction or a transgenic VEGF gain-of-function system could be used to modestly increase circulatory VEGF. This would protect against capillary loss, compromised perfusion, and decreased tissue oxygenation that occurs due to aging. Inflammation decreased metabolic flexibility, endothelial cell senescence, and other signs of aging in mice treated with VEGF. In contrast, conditional expression of recombinant sFlt1 prompted the formation of these undesirable age-related traits by impairing VEGF activity in endothelial cells. Decreased abdominal fat deposition, decreased liver steatosis, decreased sarcopenia and good restoration of muscle-generating force, decreased osteoporosis, decreased kyphosis, and decreased stress of spontaneous tumors, were observed in VEGF-treated rodents, which consequently had a higher survival rate and improved health [68,69].

### 4.5. Diet That Improves Vascular Function to Delay Aging

A diet that contains low methionine levels or in other words a methionine-restricted diet improves vascular function via forming new blood vessels and thus it can help to extend life expectancy. These studies show that these changes may be due to improved vascular function. One benefit of a methionine-restricted diet is new blood vessel formation that is promoted by gas production, i.e., hydrogen sulfide. These findings suggest that there is a link between a methionine-restricted diet and angiogenesis. To determine this, scientists have researched mice. They fed rodents a methionine-restricted diet that had lower amounts of cysteine. Protein-rich foods include meat, dairy, nuts, soy, and avocado. These protein-rich foods are enriched with these two amino acids (methionine and cysteine). The mice showed an increase in formation of blood vessels in skeletal muscle after two months compared with mice that were fed a normal diet. Another triggering factor of angiogenesis is hypoxia. These studies should help in the future to enhance angiogenesis of vascular aging or to inhibit angiogenesis for the prevention of the formation of tumors [70].

### 4.6. Role of FOLR1 in Aging

FOLR1 stands for folate receptor 1 or folate receptor alpha which is present on the q arm of chromosome no. 11. It produces a protein that may either exist in soluble form or anchor to the membrane through a glycosyl–phosphatidylinositol linkage [71]. It helps in the transportation of folic acid in the cells and thus slows down the aging process. Mutations, on the other hand, can cause functional changes in folate absorption in the cells. In addition, neurodegenerative disorders have been observed due to mutations in these genes, which is also a sign of aging. This gene also helps in the upregulation of Yamanaka factors [octamer binding protein 4 (Oct4), sex determining region Y-box 2 (Sox2), Kruppel-like factor 4 (Klf4), and cellular myc (cMyc)] [72].

## 5. Intake of Folic Acid Leading to a Slow Aging Process

Folic acid’s proper intake via diet helps the body to produce new cells and as well as maintain them. It also helps to prevent any changes in the DNA that may be carcinogenic when exposed to the sun. Folic acid helps in detoxification of the body by reducing oxidative stress levels in the skin. It is involved in the development of healthy skin cells and their rejuvenation by increasing the production of collagen; it slows down the aging of the skin, fine lines, and wrinkles, and thus makes a person look younger [71,72]. In age-related cognitive and memory decline, folic acid helps as well. Taking the recommended levels of it can prevent brain diseases such as Alzheimer’s and dementia, etc. It was reported that elderly people who took folic acid supplements had the memory and cognitive abilities of someone 5 to 6 years younger than their age. Moreover, in terms of muscular activities, research has shown that intake of adequate levels of folic acid improves muscle skills equal to the one who’s 2 years younger than their age [73]. Another important thing when getting older is the loss of hair and hair color. Hair pigment (melanin) is lost when there is an increased production of large-sized RBCs due to a deficiency of folic acid leading to premature greying of hair. Its intake improves hair thickness, avoids hair loss, and promotes hair growth. As hair grows, melanocytes stop producing melanin leading to grey hair; having partial melanin, and white hair; with no melanin, but vitamin B12 aids in restoring healthy hair and its color [74].

### 5.1. Effect of Deficiency of Folate in Elderly People

Folate, which is also known as vitamin B12 or B9 has several negative impacts if the body is deficient in it. This is mainly due to the intake of unhealthy and unbalanced diets having low dietary folate. The effects of deficiency of folate are the production of larger-sized abnormal RBCs having improper functioning leading to megaloblastic anemia [74], fatigue, weakness, neurological disorders, and loss of hair color and hair. In elderly people, deficiency causes early aging along with extreme tiredness or lack of energy, paresthesia, loss of muscular functions, and depression and anxiety [73,74]. These are all the symptoms that are common, as well as found in older people; therefore, if the recommended dosage of folate is met these symptoms can be reversed.

### 5.2. Dosage of Folic Acid by FDA

It is recommended by the FDA that seniors should consume 400 micrograms of folic acid each day to be in shape and remain active [74].

### 5.3. Folate-Containing Foods

**Figure 5:**
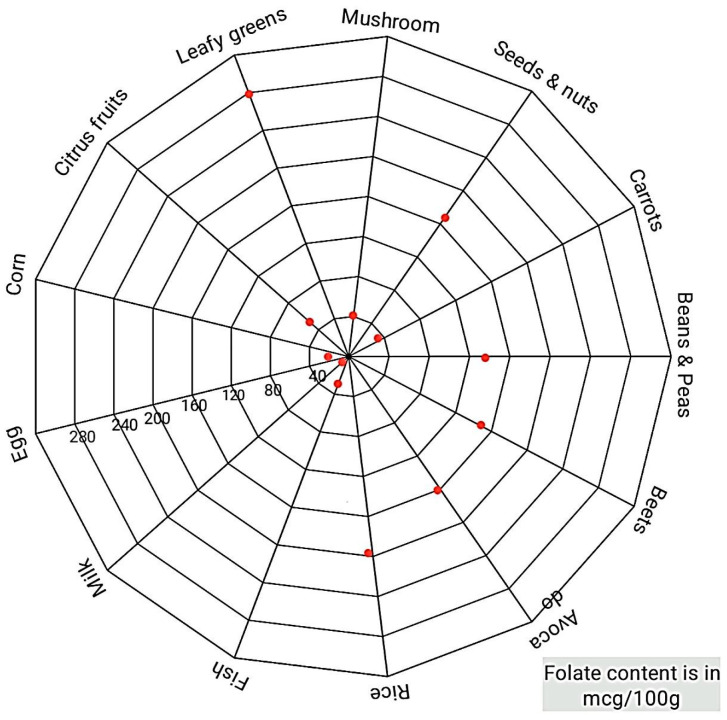
Folate-containing foods, the maximum folate is present in leafy green vegetables, followed by seeds, nuts, and rice; mcg/100 g [75].

Several foods contain folate naturally. Some of these are green leafy vegetables including spinach, broccoli, asparagus, etc., beans and whole grains, seafood, meat, dairy products, fresh fruits, and juices [75]. Figure 5 shows folate-containing foods.

## 6. Other Genes Affecting the Expressions of FOLR1 and VEGF

### 6.1. GATA6 Gene

GATA6 induces the aging responses and when mesenchymal stem cells (MSC) age, the GATA6 transcriptional factor starts to be produced in high amounts. However, if cellular reprogramming is achieved and old mesenchymal stem cells are rejuvenated, the aging effects can be reduced [76].

Yamanaka factors have the potential to reset the epigenetic markers to their original forms and thus are involved in cellular reprogramming. These factors are a mixture of four reprogramming molecules, Oct4, Sox2, Klf4, and cMyc, whereas, in mice, these factors showed muscle regeneration [40,77]. Researchers found that in response to folic acid, folate receptor alpha activates the transcription process by translocating to the nucleus where it binds the cis-regulatory elements of Oct4, Sox2, and Klf4. This in turn activates the downstream targets of Oct4 such as Trim71 (Tripartite motif containing 71) and downregulates miR138 and miR-let-7 levels, which target Oct4 and Trim71, respectively. This suggested a novel pleiotropic role of folate receptor alpha (FOLR1) [76,77]:

(a) direct activation of genes associated with maintaining stem cell characteristics (Oct4, Sox2, and Klf4 genes);
(b) repression of miRNA biogenesis associated with these genes or their effector molecules, as demonstrated for Oct4 and its effector target Trim71.

### 6.2. CBS Gene

**Figure 6:**
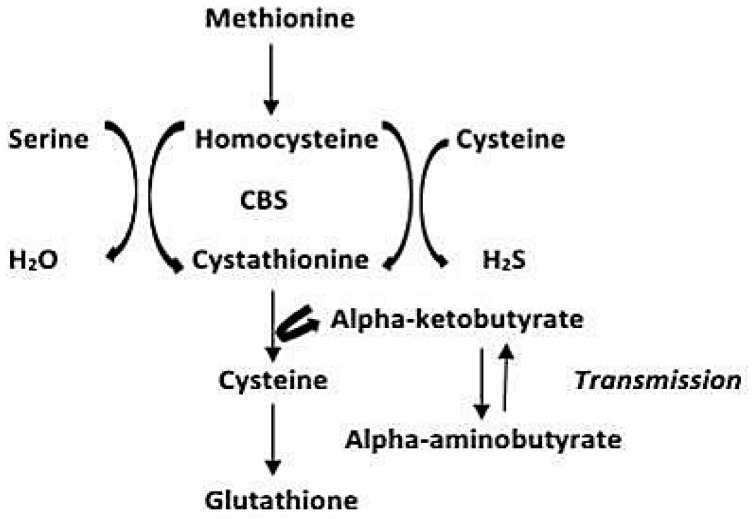
The pathway showing the metabolism of homocysteine to cystathionine by using vitamin B6, and resultant formation of glutathione [69].

CBS stands for cystathionine beta synthase, a key enzyme in homocysteine metabolism, encoded by the CBS gene. This enzyme is involved in the conversion of homocysteine to cystathionine by using vitamin B6 [69]. Figure 6 shows the pathway of metabolism. A study revealed that a decrease in the activity of CBS enzyme leads to a state called hyperhomocysteinemia, abbreviated as HHcy; therefore, the methylation of promoter and the polymorphisms of CBS genes can affect the efficacy of HHcy and using folate the therapy was conducted against low levels of Hcy [78]. Moreover, an increase in homocysteine levels also causes age-related memory loss and cardiovascular disorders. Also in elderly people, homocysteine levels were observed to be higher than in adults [79].

Therefore, a close association between CBS and the FOLR1 gene exists, and thus somehow indirectly this gene is also involved in the body’s functioning and aging. Moreover, an increase in homocysteine levels also causes age-related memory loss and cardiovascular disorders. Also in elderly people, the levels of homocysteine levels are observed higher than the adults [24]. FOLR1 however, downregulates Hcy levels. Therefore, a close association between CBS and FOLR1 gene exists, and thus somehow indirectly this gene is also involved [80].

### 6.3. CISD2 Gene

CISD2 gene stands for CDGSH iron–sulfur domain-containing protein 2. It is involved in glucose/energy metabolism. In this metabolic pathway, a total of 307 genes are involved. SIRT1 is one of them [81]. SIRT1 is said to be associated with longevity. Moreover, research has shown that this CISD2 gene exists on chromosome 4 on a locus that correlates to genetic differences in the lifespan. This gene maintains the mitochondrial working and thus regulates cell division and growth. If the levels change then this gene induces age-related disorders such as Alzheimer’s disease. In mice, research showed that absence of CISD2 is involved in premature aging; therefore, by regulating this gene product’s levels in humans, it was said that aging can be delayed [82].

### 6.4. SIRT1 and SIRT6 Genes

These are members of the sirtuin family and have roles in processes such as regulation of obesity, aging, inflammation, and energy metabolism. SIRT6 genes deacetylate lysine 9 on the N-terminal tail of histone H3 (H3K9Ac) to modulate telomeric chromatin and gene expression. In mice, a decrease in SIRT levels resulted in age-related genomic instability, metabolic disturbances, and degenerative phenotypes [83]. Reduced SIRT levels are involved in diabetes and obesity mostly observed in elderly people. SIRT1, however, is said to be associated with delayed aging. SIRT 6 can decrease the level of VEGF and angiogenesis by inhibiting the expression of Hif-alpha in tumor development [84].

## 7. Associated Diseases

### 7.1. VEGF-Associated Diseases

**Figure 7:**
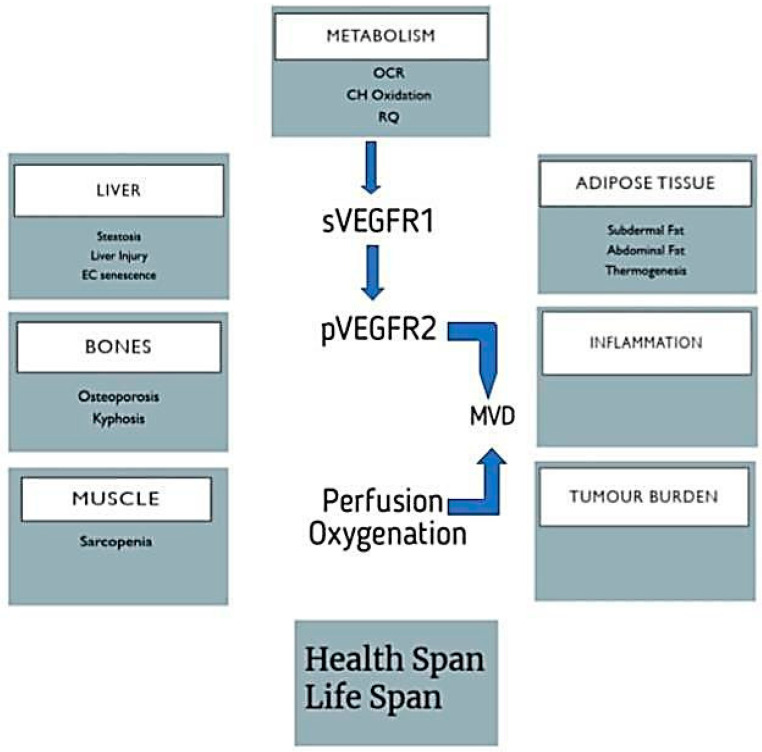
Role of VEGF in different diseases such as liver injury, osteoporosis, kyphosis, sarcopenia, tumor burden, and inflammation [80,81,85,86].

Dysregulation of VEGF causes cancer, vascular aging, and heart diseases as discussed above [22,23]. Figure 7 shows some other problems that are experienced in different parts of the body due to VEGF’s problematic regulation [80,81].

### 7.2. FOLR1-Associated Diseases

When the FOLR1 gene undergoes some mutation or due to genetic issues show less or no expression in the form of protein enzymes, then several neurodegenerative disorders take place. These include neurodegeneration due to cerebral folate transport deficiency and neural tube defects in infants. If exposed to slightly low pH after the conformational change induced by receptor endocytosis, the affinity for folate within the membrane decreases, and thus folate is released. Because of this, normal cell and embryonic proliferation and development, respectively, can not occur [85].

## 8. Nanotherapeutics and Aging

The main causes of aging and most neurological illnesses are increased oxidative stress and subsequent systemic inflammation. Thus, antioxidant supplementation is seen as a viable treatment for brain diseases and aging. These supplements most frequently consist of food-grade antioxidants made from plant extracts. Often-utilized phytochemicals for possibly reducing cellular damage brought on by free radicals include curcumin, resveratrol, catechins, and quercetin [86]. However, these phyto-antioxidants’ weak solubility and stability restrict their absorption in the gastrointestinal tract, lowering their bioavailability. In preclinical studies, these nanotherapeutics demonstrated improved solubility, stability, and permeability across gastric mucosa compared to standard formulations. Additionally, they have shown improved tissue targeting, increased bioavailability, and a longer half-life with reduced detrimental effects. The drugs’ pharmacokinetics may be changed with aging and can affect the efficacy of the drugs in older people. There is an overview of the broad changes in drug absorption, distribution, cellular uptake, and metabolism facilitated by nanomaterials [87]. Advantages and the areas of overlap for nanotherapeutics found by the researchers [88] are given in Table 1.

With delayed uptake time, reduced absorption of weakly basic medications, and impaired uptake of certain soluble transporter pharmaceuticals in older individuals, nanotherapeutics provide the potential to significantly improve therapeutic drug absorption. These advantages are possible because nanotherapeutics are widely absorbed by a variety of soluble transporters and exploit the mechanisms of clathrin endocytosis and micropinocytosis to boost drug bioavailability. Moreover, nanotherapeutics show typical and very selective distribution and liver clearance [88]. Different drug formulas are being used successfully against aging agents such as acetylcholinesterase, which provides an effective treatment against damaging cell particles. A few protein molecules such as occluding, junctional adhesion molecules, and Claudins are found to be involved across the blood–brain barrier in slowing the aging process. The bond-making ability of lipophilic NPS plays an essential role in the commute of drugs through intracellular pathways. Another chemical, melatonin-loaded chitosan, also has successful properties against cancer-causing cells of the body which lead to enhancing the aging process in the body. Apolipoprotein, a subclass of lipo-protein, shows an associated relationship with polymorphic alleles having genetic effects inhibiting aging for a prolonged time. In the study, it was observed that parasympathomimetics manufactured by USA companies also play a role as nano-therapeutics agents in delaying cell-damaging with age [87,88].

## 9. Nanoparticles: As Drugs and Vaccine Delivery Systems via the Nasal Cavity

The nose has great importance and is positioned under and in the middle of the eyes. The nostrils open into the esophagus and trachea and are the back entrance. Oppositely nares have exterior openings. The nose is the main and most important opening of the respiratory tract. It plays a basic role in respiration. It performs many functions such as warming, moisturizing, and filtering air when it passes through the nose. It also plays an important role due to the presence of olfactory organs [89].

### 9.1. Nasal System

The nasal cavity has a typical shape, and it extends from the bony plate to a pointing cranium. The bony plate is made up of seven bones. Due to this system, a chamber is created which is 7.5 cm and 5 cm in length and width. The nose is separated into two parts with the help of the nasal septum [90]. The vomer is also present in it and is located anteriorly to the ethmoid bone and posteriorly to the septal cartilage. The nasal septum is extended from the nares to the nasopharynx. A soft plate, hard palatine bone, and tissue flap form the roof of the mouth and floor of the nose. The soft plate creates a system to avoid being stuck in food at the nose back. After food passage, the soft plate moves upward and clears the way for air. This makes the capacity to breathe from the nose as well as from the mouth but breathing has some advantages such as humidity, filtration, and heating of the air [91,92]. 

The nasal cavity is known for transferring infection, due to its larger site for entry of the pathogens into the body without resistance. This site of the body possesses a peak permeability factor with less enzymatic activity. The microvillus region of the nose allows the capacity for vaccine particles to be absorbed and digested through the nasal cell wall. The first line of defense is humoral immunity involved in the production of B-cells by immunoglobulin A (IgA), which is available in the nasal cavity in a dimeric shape; the defense cells of lymphoid tissues are used in the activation of cellular and humoral immunity in the body. The main achievement of vaccination is to keep B and T cells active against antigen particles [93].

The components involved in the manufacturing of nanoparticles are polymers including polylactide and polyglycolide. Few other particles such as copolymers and polyacrylates with natural chemicals such as gelatin and collagens are included. These polymers have been used for animal and human health. A form of modulating polymers can be used for therapeutic capacity of nanoparticle application to humans up to the desired level. 

**Figure 8:**
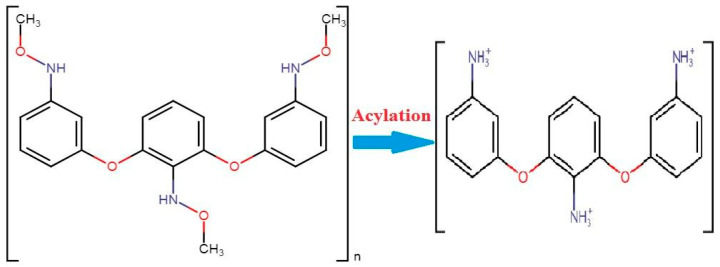
The conversion of chitin into chitosan under the process of acylation [95].

Another polymer, polyethylene glycol, is important against corona as a nasal protein. The stable character of this chemical makes it more attractive for nasal application while escaping from enzyme interactions. Chitosan polymer creates a barrier between antigen and body humoral immunity while inhibiting the escape of antigens from body immunity. A mixture of chitosan enhances the absorption power of the nasal cavity. These techniques may be helpful in the application of vaccines. Researcher De-Haan and his colleagues have successfully delivered a vaccine using liposomes [94]. They proved the efficacy of using of liposomes against the influenza virus with vaccine subunits. An experimental application of chitosan was performed against hepatitis B. This viral disease kills more than 25% of the world’s population every year. A vaccine is being produced against it, but it are manually applied, which also causes pain so the administration is not suitable. For that reason, nanoparticle applications have been introduced to treat life threatening diseases. When chitin combines with acylation it results in chitosan. These particles have a size of 250 nm to 350 nm with more than a 28,000 molecular weight. A dry form of the chemical tripolyphosphate has been used in the purification of chitosan manufacturing during the acylation of chitin (Figure 8) [95].

A study shows that the nanoscale method of vaccine delivery is more efficient and effective than oral uptake of medications. A huge number of novel therapeutic chemicals, including proteins, plasmid DNA and peptides have been developed. Nasal medications are facilitated by (NALT) nasal-associated lymphoid tissue, which holds aerosols of the vaccine within its epithelial surface [96]. The oral intake of medicine or chemicals is a little complicated by low chemical absorption rate, less bioavailability, low enzymatic breakdown, and some other substituent effects on the digestive system of the body. This draws the focus of chemical industries and pharmaceutical companies toward nasal ingestion of medicine. For nasal inhalation of medicine, a low molecular weight of medicine is required, especially at that time when rapid action of the medicine is required [97].

**Figure 9:**
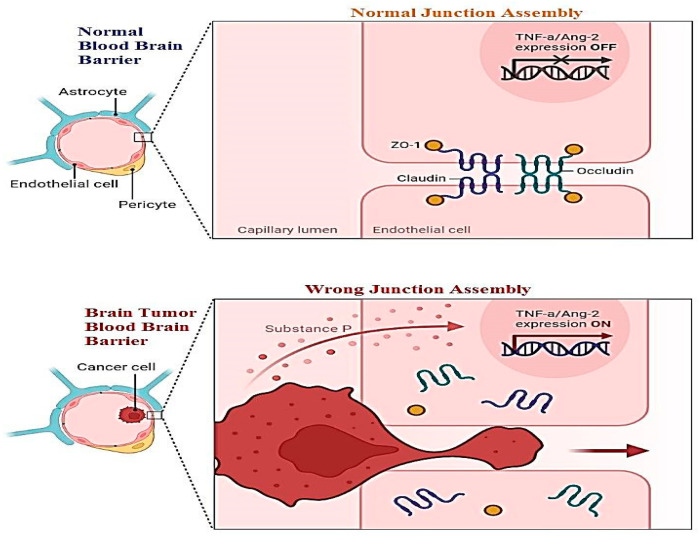
Difference between normal blood–brain barrier showing normal occluding and claudin protein junctions, and brain tumor blood–brain barrier disturbing occluding and claudin by invading the normal cell membrane [83,98,104].

A new challenge must be health-related staff to manage the blood–brain barrier. This layer relates to all the parts of the brain including blood capillaries. Direct medication from the nose to the brain is a novel methodology of treatment. For this purpose, two pathways have been introduced: the first is trigeminal, and the second is an olfactory passageway for treatment with an application of nanoparticles. Drawbacks of these techniques including cell damage and the inflammatory response have been observed. Nanoparticles are best used for the delivery of DNA vaccines. Vectors used for the transportation of DNA vaccines are lentiviral vectors and adeno-associated-viral-vectors (AAVV). An alternative to viral vectors are nanoparticles highlighted for the safe delivery of DNA vaccines. Figure 9 shows the difference between the normal blood–brain barrier and the brain tumor blood–brain barrier. It shows swelling and clotting of blood cells inside the layer which resist cerebrospinal fluid (CSF) transport of material across the wall [98].

**Figure 10:**
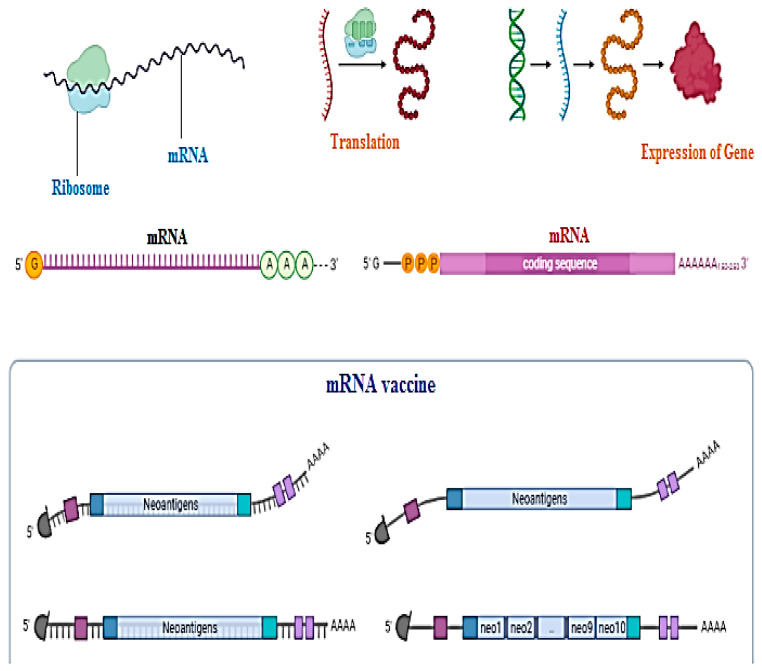
Entry of DNA vaccines into the nucleic acid region for their activation as compared to mRNA vaccines for which the cytosol area is enough for activation [102,103].

Messenger RNA or DNA vaccine techniques are used to make clones of antigens just to trigger the body’s immune response to become immunized against disease [99,100,101,102]. Not a single DNA vaccine has been approved by the FDA, especially for human use, but in the future, they could be utilized for the service of mankind. Nanoparticles are a hot issue for application of DNA vaccines today. The beneficial aspects of DNA vaccines as compared to traditional vaccines are that they create no issues with recombinant proteins such as wrong folding of proteins or becoming reduced during structural formation, its low cost of synthesis, and that it can trigger both lines of immunity, cellular as well as humoral. Chemical changes in the coding gene region, poly-A-tail, can provide an edge to structural stability and authenticity of DNA vaccines. DNA vaccines need to enter the nucleic acid region for their activation as compared to mRNA vaccines for which the cytosol area is enough for activation as shown in Figure 10 [102,103]. 

### 9.2. Zx+10a0 Nasal Vaccination: Why Nanoparticles?

**Figure 11:**
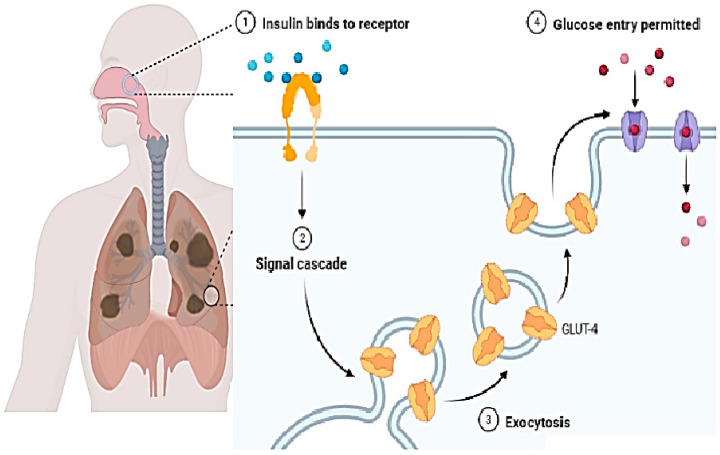
Inhalation of vaccine through the nasal cavity, and its mechanism inside the body in immune cells [103,105,106].

In present times, the nasal route is gaining growing interest and some drugs having low molecular weight have been approved and are available in the market. Butorphanol is used for pain relief and is applied via the nasal route. For cryptorchidism, luteinizing hormone-releasing hormone (LHRH) is used and is taken via the nasal route. Many other drugs having low molecular weight are also being applied via the nasal route [103]. In the case of larger molecules, (for example, proteins) this route is not used frequently. This is due to the drug absorption problem. Hence, further strategies should be developed to improve drug absorption [104]. Figure 11 shows the inhalation of a vaccine through the nasal cavity and its mechanism inside the body in immune cells.

The mass cut-off value for the permeation of molecules via the nasal system is approximately 1000 Da. Hence, larger molecules are not allowed, and absorption enhancers are required for this. To overcome the problem, nanoparticles are the best options as they are successful adjuvants that are used as the delivery system or/and immune modulators for the application of vaccines [105]. They also have a beautiful attribute in that they can protect against antigenic degradation from proteolytic enzymes, hence ensuring drug delivery at the cellular level. An interesting fact is that they can pass the mucus and directly interact with the mucosal cells and enhance immunity activation. One can also change the attributes of the nanoparticles to obtain the results of one’s own interest. Due to their unique small size, they also show mimicry with viruses [106]. Due to their small size, they can pass through the mucus barrier. Therefore, they have high cellular interaction. The size of nanoparticles ranges from approximately 20 nm to 80 nm. This wide range is administered in a dose making them interesting to use. They also have a hydrophobic nature which helps them pass the mucosa. These important features of nanoparticles make them a strong candidate for use in drug delivery via the nasal cavity [107].

### 9.3. Features of the Nose for the Delivery of Drugs and Vaccines

To understand the phenomenon of immune system triggering during the delivery of a drug, it is important to compare rodent with human noses. In the case of rodents, nose-associated lymphoid tissues (NALT) are lymphoid tissues [108]. They are present at the dorsal nose duct bottom. These are bell-shaped and paired tissues and lymphoid tissues are accumulated in their formation. This process is completed in around 5–8 weeks after birth. In human beings, NALT is composed of tonsils and adenoids. This is an important feature of the human mucosal immune system. “Waldeyer’s ring” was identified in 1884 by Waldeyer. It is formed of adenoid or nasopharyngeal tonsils in addition to “paired palatine tonsils”, “the paired tubal tonsils” and lingual tonsils [109]. 

Tonsils are in the lamina propria of the pharyngeal wall, and these are secondary lymphoid organs. When observed under a microscope, the surface of the tonsils is made up of various epithelial linings and are called crypts. These crypts deeply penetrate the lymphoid tissues. They play an important role in the respiratory immune system by increasing the surface area of the tonsils [110]. They are specially designed to trap foreign materials. The histology and anatomy of both human and mouse nasal cavities are different. In the case of mice, single-layer epithelium makes up the murine respiratory epithelium. Along with this, in the turbine portion, columnar epithelial cells are present. The olfactory epithelium of mice is covered with “pseudostratified columnar epithelium”. In the case of human beings, there is no single epithelium layer. Pseudostratified columnar epithelium covers both the olfactory and respiratory surfaces. Notably, in nasal epithelium cells and the upper airway, tight junction (TJ) molecules are present. Due to the presence of these structures, permeability of the human nasal epithelium layer is poor [111].

#### Activation of the Mucosal Immune System by Nanoparticles

There are two main components of the mucosal immune system keeping in view the anatomy and structure. These two components are [112,113]:

(1) effectors sites;
(2) inductive sites.

In inductive sites, an antigen-specific immune system is activated, and it is composed of “organized mucosa-associated lymph tissues”. The effector sites are usually associated with “cell-mediated response” and with the production of antibodies [114]. NALT is a site of both humoral and cell-mediated activation. In NALT all those cells that are necessary for immune response are present. Here B cells, T cells, dendritic cells, and antigen-presenting cells (APC) are present. All these sites are directly associated with the inductive sites through the mucosal immune system for antigen-specific immune response. This system works as the first line of defense at the mucosal surface [115].

M cells are also present in NALT. Their basic work is to screen antigens to recruit potential pathogens for immune response. M cells perform two very important functions [116]:

(1) barrier maintenance;
(2) mucosal immune response initiation.

M cells in NALT are the key players in nanoparticle uptake and producing IgA antibodies. Mucus is the epithelial layer and is the first barrier that nanoparticles must face. The epithelial layer not only acts as a barrier line but it also secretes cytokine and activates an innate immune response [117]. 

The adaptive immune response is initiated by the APC with the help of MHC. Dendritic cells (DC) can move from NALT to lymph nodes and stimulate the helper T cells or cytotoxic T-cell response. In this process, with the involvement of NK cells and eosinophils at the end, the cell is lysed [118]. 

### 9.4. Nasal Drug and Vaccine Delivery and Nanoparticles

There are four major types of nanoparticles that have been prepared and are being used for drug and vaccine delivery via the nasal cavity [112,113]:

(1) polysaccharide nanoparticles;
(2) protein nanoparticles;
(3) lipid nanoparticles;
(4) polymers nanoparticles.

Different laboratories around the world use different methods of administration. Some information about the dose, volume, antigen concentration, number of administrations, and or without the process of anesthesia are summarized in Table 2. Next we discuss the different types of nanoparticles used in the delivery of drugs and vaccines [119]. 

#### 9.4.1. Polysaccharide-Based Nanoparticles

Polysaccharide nanoparticles have been widely used as delivery systems thanks to their important property of “biocompatibility”. 

#### 9.4.2. Chitosan Nanoparticles

Chitosan is an important polymer of N-acetylglucosamine and glucosamine. It is derived from the partial deacetylation of chitin. Chitin is abundantly present in shellfish. It has been used as an absorption enhancer in many drugs. It is mucoadhesive and is soluble at acidic pH [120]. Its efficacy is dependent upon the degree of deacetylation. The nature of these nanoparticles is mucoadhesive. Due to this property, they have more retention time, and their clearance is delayed.

Therefore, the retention time in the mucosa is also increased [121]. This also increases the contact time between formation and NALT. Due to the mucoadhesive nature of chitosan nanoparticles, the residence time of the administered solution could be quadrupled, thanks to this unique property.

They also have adjuvant properties and are very successful in the case of different protein antigens such as recombinant anthrax, influenza, hepatitis B, and ovalbumin. When experiments were conducted on a mice model, they showed an improved version of humoral (IgG) and mucosal (IgA) responses. These nanoparticles were loaded with toxoid of tetanus, and they triggered both IgA and IgG production [122,123,124,125,126,127,128,129,130,131,132].

#### 9.4.3. Starch Nanoparticles

Starch is a polysaccharide found in nature, and it is composed of amylopectin and amylose. This carbohydrate is abundant in the amyloplasts of plants. Here it works as an energy reserve. As a result of partial starch hydrolysis, maltodextrin is obtained. It is also used in the synthesis of nanoparticles [133]. These are biodegradable and have a wide range of applications in drug delivery and nano vaccines. In an experiment, influenza virus antigens were encapsulated in a propyl acrylic acid and starch mixture. As a result, an IgG-specific response was achieved. In the case of intranasal vaccination, maltodextrin is a promising mucoadhesive polysaccharide nanoparticle. When it was loaded with the antigen of hepatitis B, it showed more mucosal, humoral, and cellular immune responses than did free antigens [134]. 

### 9.5. Polymer Nanoparticles

Co-polymer of lactic acid and glycolic acid (PLGA) is one of the most important synthetic polymers for nanoparticle preparation. They are biodegradable and biocompatible, thanks to their unique properties. Polylactic acid (PLA) could be used for nanoparticle preparation to delay the rate of delivery for drugs having low molecular weights. Glycolic acid has been added to PLA to formulate PLGA [134]. Now, a very wide and unique variety of PLGA polymers is available in the market. Different ratios of moles have been used in them for the formulation of polymers having unique and different properties. They have either an acid or ester as a terminal group, which determines their hydrophobicity. PLGA has the capability of antigen encapsulation in the structure of the matrix, or they can adsorb proteins on the surface. Encapsulation of antigens allows the controlled and sustained release of antigens. This leads to an improved response of the immune system. If PLGA is coated with polyethylene glycol (PEG), it favors the passage of antigens and drugs from the mucosa. Naturally, PLGA is negatively charged but they can be converted to be positively charged by adding cationic ligands (e.g., chitosan). PLGA nanoparticles also play their role in increasing drug permeability into the mucosa. PLGA nanoparticles, when conjugated with transferrin, showed good gene delivery results via the nasal cavity [135]. PLGA nanoparticles also have a wide range of applications in veterinary medicine. PLGA particles were loaded with rE2 glycoprotein and showed good results when administered via the nasal cavity as compared to oral administration. So far, most polymer nanoparticle studies have been limited to preclinical development [136]. 

### 9.6. Lipid-Based Nanoparticles

They have a hybrid structure between liposomes and polymer nano-capsules. Triglycerides of the medium chain form their core which is surrounded by polyethylene glycol (PEG) and lecithin. They can be prepared in a solvent-free environment, which makes them unique. Another important property is their high stability as compared to some other lipid-based nanoparticles [136]. In one study, toll-like receptors (TLR) were combined with lipid nano-capsules and prepared for mucosal drug delivery. When applied in mice, it showed long-living T cells in the lungs and vaginal mucosa. These are powerful tools for mucosal drug delivery and vaccination but very little work has been published on this topic so far [137,138,139]. 

#### Immune Stimulating Complexes (ISCOMs)

ISCOMs are about nm in size and have an open-caged spherical shape. They are usually made up of phospholipids, cholesterol, and Quil A (usually extracted from the bark of a plant named Quillaja Saponaria). The hydrophobic portion of Quil A is made up of carbohydrates while the hydrophilic portion is made up of quillaic acid, so it is amphiphilic [140]. Due to the presence of glucuronic acid at their surface, they are negatively charged. Some cationic derivations of them have also been prepared. Ovalbumin-ISCOMs were prepared, and they induced mucosal and systemic immune responses in mice. In the case of larger animal models, intranasal administration of ISCOMs showed stimulation of IgA production. They showed good results in the case of bovine respiratory syncytial virus. There are very few brands of ISCOMs available in the market for nasal vaccines and drug administration [141]. 

### 9.7. Protein-Based Nanoparticles

Protein nanoparticles are usually proteosomes. These are usually prepared from the purification of outer-membrane proteins obtained from Neisseria meningitides. These delivery systems are hydrophobic. These can be used for drug delivery and subunit vaccine administration via the nasal cavity. In research conducted by Plante and co-workers, a proteosome-based influenza vaccine was administered in mice. The vaccine induced IgA and IgG-based immunity and protected against the viral challenge [142]. Vaccines based on proteosomes were tested against shigella and influenza. They successfully induced nasal secretory mucosal antibodies (sIgA). Lipid moiety and antigenic peptides are fused to form lipid core peptides. A vaccine against streptococcus was prepared based on B cells and T cell epitopes fusion with a tandem of C16-lipoamino acid. Studies have also shown that a combination of PLGA or other polymer-based nanoparticles with protein nanoparticles can increase the efficiency of vaccine and drug delivery [143]. 

## 10. Conclusions

The current study focused on octogenarians for genetic analysis of aging, physical parameters of aging genetics, and the application of nanotherapeutic parameters for drug delivery systems. VEGF serves as a key mediator involved in angiogenesis. Insufficient signaling of VEGF causes vascular and multiple organ aging. Maintenance of a sufficient level of VEGF can serve as a factor to be used as a gero-protection. Methylated restricted diets can delay aging by enhancing vascular function; on the other hand, high protein diets including meat reduces vascular function as they have a higher proportion of methylated amino acids. FOLR1 also plays an important role in the aging process. Several genes play a significant role in the upregulation or downregulation of the FOLR1 gene. Symptoms of aging can be delayed by using advanced techniques like gene therapy for regeneration. Through regulation of VEGF and FOLR1 levels, this research will pave the way for genetic engineering to increase healthy life spans in humans. The nanotherapeutics application through drug delivery systems, most particularly through the nasal cavity, may aid in delaying the aging process, and its subsequent effects have been discussed in the current study. The local inflammatory profiles in the liver and blood were found to be a challenging task for the application of nanotherapeutics in older individuals. The effectiveness and long-term toxicity of nanotherapeutics in elderly people may fluctuate significantly depending on the extent of complement, IgG, and protein binding variations. Nanoparticles could revolutionize the world as they hold tremendous potential both in drug delivery and mucosal vaccines. Despite the great potential of nano vaccines, results of clinical trials have been very sparse and not as promising as they have been in animal models. To better understand nanotherapeutic applications, it is important to carry out further investigation in aged animal models and relevant metabolic pathways in older people.

## Figures and Tables

**Table 1:** Summary of the changes with aging on drug deposition and the effect on nanotherapeutics.

Features	Changes with Aging	Effects on Nanotherapeutics (NPs)
**Absorption**		
Gastric acid	Decreased secretion	Reduced degradation
Microbiota	Reduced diversity and leaky gut	Increased absorption time
Gut motility	Delayed gastric emptying	Potential increase in toxicity
Injectable permeability	Injection absorption unaffected	Injectable NPs are unaffected
**Distribution**		
Plasma proteins	Reduced circulating albumin	May reduce protein corona
Body composition	Decreased water and increased fat contents	May reduce the distribution of water-soluble NPs and increase the distribution of fat-soluble NPs.
**Cellular uptake**		
Phagocytosis	Reduced clearance and high circulating inflammatory markers	Unaffected NPs uptake
Endocytosis	By LSECs is slightly reduced	Largely unaffected
Transcytosis	By LSECs is unaffected	Unaffected
Passive uptake	Decreased due to pseudocappilarization	Reduced NP uptake
**Metabolism**		
Phase I	Reduced due to hepatic blood flow	Prolonged circulation time
Phase II	Reduced	Unaffected

**Table 2:** Nanoparticles for nasal vaccination, immunization parameters, and immune response.

Type of Particles	Particles Characteristics		Model	Immunization Parameters			Immunity Response	Reference
	Size (nm)	Z (mV)		Admi.	Ag Dose (µg)	Anes.		
Chitosan	300–680	26	Mice	3 × 15 µL	2.5	Yes	h, m	(Bento et al.) [120]
	80	14	Mice	2 × 10 µL	10	Yes	h, m	(Pawar et al.) [121]
Maltodextrin	70	38	Mice	3 × 12 µL	10	No	h, c, m	(Debin et al.) [122]
Chitosan PLGA	500 nm–2 µm	-	Cattle	1/2/3 mL	10–15	-	h, m	(Pan et al.) [123]
Liposomes	30–100	-	Mice	3 × 50 µL	-	-	c	(Ninomiya et al.) [124]
ISCOMs	40–50	-	Mice	2 µL	2	Yes	h, m	(Cibulski et al.) [125]
Polystyrene	300–390	-	Mice	3 × 20 µL	10	-	c	(Misstear et al.) [126]
PLA microparticle	-	-	Mice	10 × 50 µL	-	Yes	h	(Rice-Ficht et al.) [127]
Lipopeptide	150–1000	-	Mice	3 × 10 µL	40	-	h, m	(Zaman et al.) [128]
Dextran	140–310	−38:39	Mice	3 × 10 µL	10	No	h, m	(Marasini et al.) [129]

Abbreviations: Zeta potential (Z), Antigen (Ag), Anesthesia (Anes.), Administration (Admi.), the response of antigen is indicated as h—humoral, m—mucosal, c—cellular, Polylactic-co-glycolic acid—PLGA, polylactic acid (PLA).

## Data Availability

Not applicable.
